# The Role of Immune Markers in Predicting Infectious Complications in Children with Congenital Heart Defects

**DOI:** 10.2174/0115733963325523250320065040

**Published:** 2025-03-26

**Authors:** Degtyareva Elena, Mwela Bupe Mumba, Prodeus Andrey, Ovsyannikov Dmitry, Kantemirova Marina, Alekseeva Olga, Kudlay Dmitry, Kim Alexey, Nefedova Inessa, Rogova Tatyana, Tumanyan Margarita, Korsunsky Iliya

**Affiliations:** 1 Federal State Autonomous Educational Institution of Higher Education “People's Friendship University of Russia” named after Patrice Lumumba, Moscow, Russia;; 2 State Government-Funded Healthcare Institution of the Moscow Region “Research Clinical Institute of Childhood of the Ministry of Health of the Moscow Region,” Moscow Region, Mytishchi, Russia;; 3 I.M. Sechenov First Moscow State Medical University (Sechenov University), Moscow, Russian Federation;; 4 NRC Institute of Immunology FMBA of Russia, Moscow, Russian Federation;; 5 Federal Government- Funded Institution “National Medical Research Center for Cardiovascular Surgery named after A.N. Bakulev,” Ministry of Health of the Russian Federation, Moscow, Russia;; 6 “Rassvet” Medical Center, Moscow, Russian Federation

**Keywords:** Congenital heart defect (CHD), immunodeficiency disorder (IDD), primary immunodeficiency (PID), secondary immunodeficiency, immunity studies, TREC, KREC

## Abstract

The literature review presents data from a limited number of available studies conducted over the last two decades on immunological deficiency in congenital heart defects (CHDs), which is the cause of frequent infectious complications before and after cardiac surgery. Several studies based on screenings at various levels indicate the presence of primary and secondary immunodeficiency in CHDs, in particular about 13 genetic syndromes in which CHD is combined with immunodeficiency. The available data suggests a greater severity of immunological disorders in patients with critical CHDs, cyanotic CHDs, and conotruncal defects with T-cell dysfunction and deficiency of immunoglobulins (especially the IgG class, mainly IgG4) than in patients with shunts and obstructive defects. To identify defects in the T- and B-cell components of the immune system, quantification of the DNA of T-cell receptor excision circles (TRECs) and K-deleting recombination excision circles (KRECs)-by-products of the maturation of T- and B-cell receptors-has proven helpful in the world practice of neonatal screening. It allows the evaluation of a number of functionally mature T- and B-cells. In Russia, however, its widespread use started only in 2023. Data on the use of this assay in infants with CHDs are represented by isolated case reports. In Russia, a combination of CHD and primary immunodeficiency was found in 37% of cases in the Sverdlovsk Region. We conducted our own study of 200 children with CHD; 5% of cases were syndromic forms of CHD. 48.5% of children were admitted to the cardiac surgery clinic in critical condition. A decrease in the TREC level was detected in 23.5% of cases, including all children with syndromic CHD. In the group of patients with immunological disorders, there were significantly more children with cyanotic CHD, children admitted in critical condition, and children with conotruncal defects. Infectious complications in the postoperative period (sepsis, pneumonia, tracheobronchitis, postoperative wound infection) were observed significantly more often in 47 children with reduced TREC levels compared to children with normal TREC levels (*P* = .00000, in 36% and 3.6%, respectively). The analysis of publications confirms the prognostic value of TREC and KREC screening for targeted preoperative preparation to reduce postoperative complications and decrease the risk of mortality in CHDs.

## 1 INTRODUCTION

Congenital heart defects (CHDs) are structural and functional abnormalities of the heart and blood vessels that exist from birth, even if the disorder is diagnosed much later. CHDs are a consequence of developmental abnormalities. The mortality rate in CHDs within the first year of life is at least 40%, and 70% of infants die in the first months. The primary factor affecting CHD survival is the anatomical and morphological characteristics, *i.e.* type of defect [1, 2].

CHDs are divided into critical and non-critical categories based on the defect severity and its effect on central circulation. Critical CHDs require emergency care or surgery within the first few hours, days, or weeks of life [3-5], which is facilitated by the immediate correct routing of newborns based on prenatal diagnosis of CHD and accurate urgent postnatal diagnosis [6]. Non-critical CHDs are less serious defects that can be operated on at a later age or treated with medication for a certain period of time [7]. It has been established that at least 15% of all heart defects can be caused by chromosomal aberrations, genetic mutations and be inherited [8, 9]. The number of patients undergoing surgery for CHDs in the world is increasing by 50/1,000 yearly [3]. Various types of cardiac surgery have been developed (*e.g.*, radical, palliative, hemodynamic, and endovascular) for the treatment of CHDs, depending on the clinical presentation, hemodynamics, and anatomy of the heart defects [10-14].

## 2 CONGENITAL HEART DEFECTS AND INFECTION

Timely cardiac surgery is the top contributing factor in the survival rate of children with CHDs, which has been above 90% in many treatment centers in recent decades [15]. However, surgery sometimes has to be delayed due to the intercurrent morbidity of these patients. At the same time, the risks of developing pneumonia, sepsis, and infective endocarditis in children with CHDs are significantly higher than in the healthy population, and they may lead to further complications and even the death of patients [16-18]. Woodward *et al.* 2011 associate the increased risk of infections in children with CHDs with impaired immune responses and hemodynamic disorders [19]. Thus, the severe course of bronchiolitis and pneumonia associated with respiratory syncytial virus infection, which is several times more frequent than that in non-CHD children, results in three times higher hospitalization rates and the need for treatment in intensive care units, as well as patient mortality, as established in a Canadian CHD study and others [16, 20, 21]. The incidence of pneumonia, including atypical pneumonia associated with intracellular pathogens (mycoplasma and chlamydia infections), and the persistence of pathogens may cause prolonged mechanical ventilation and acute heart failure in the early postoperative period after cardiac surgical correction of CHD [16, 22, 23]. The risk of developing endocarditis in CHD children is 40 times higher than in healthy children, and patients with cyanotic and complex CHDs before and after cardiac surgery are the main risk group for infective endocarditis, including in the neonatal period and at an early age [24]. Sepsis, a systemic infection with multiple organ damage, is a life-threatening complication in CHD children. The risks of developing sepsis and the related mortality rate in infants with CHD are significantly higher than in non-CHD infants [25]. Therefore, methods for assessing immunological disorders are crucial for assessing and minimizing the risks of infectious complications of cardiac surgery [26-28].

## 3 IMMUNODEFICIENCY DISORDERS

Immunodeficiency disorders (IDDs) are a congenital or acquired inability of the immune system to initiate cellular reactions and/or exercise humoral immunity to maintain genetically determined antigenic stability, ensuring growth and development of the body [29-32]. Primary immunodeficiencies (PIDs) are a group of genetically determined diseases that are characterized by isolated or combined defects in cellular immunity (T- and B-cells), humoral immunity, and phagocytosis. These rare disorders are characterized by susceptibility to infection and the predominance of autoimmune reactions, allergies, autoinflammation, and malignancies [32-35]. The incidence of PIDs is 5.7 per 100,000 live births [35]. Secondary IDDs can arise as a result of the exposure of an immunocompetent organism to various external factors, both infectious and non-infectious. Secondary immunodeficiencies are acquired and are more common than PIDs. The development of secondary IDDs has been described in a wide range of pathologies, including bacterial, viral (HIV), and helminth infections, emotional and physical stress, allergic and autoimmune processes, surgery, injuries, disorders associated with lymphopenia and protein loss (*e.g.*, burns, protein-losing enteropathy, *etc.*), as well as during medical procedures, radiation therapy, antibiotic therapy, the use of cytostatics and glucocorticoids, other medications, unfavorable environmental conditions, occupational hazards, quantitative and qualitative nutritional deficiencies, during periods of hormonal changes, *etc.* Secondary immunodeficiency associated with heart failure and arterial hypoxemia has been described in congenital (especially cyanotic) and acquired heart defects, including in operations using artificial blood circulation in cardiac surgery [28, 31]. When studying the relationship between IDDs and CHDs, an increased risk of emergency conditions and mortality was established in combination with thereof [36, 37].

## 4 IMMUNODEFICIENCY DISORDERS IN CHDS

The authors suggest that the susceptibility of CHD children to infection may be partly explained by an underlying immunodeficiency disorder. This was confirmed by an extensive analysis by Radford D *et al.* (1988), who found a high incidence of IDDs in children with congenital heart defects. Observing a wide range of immunoglobulin deficiencies in both defects with blood shunts and obstructive defects, as well as a significant correlation between the presence of immunodeficiency and the susceptibility to infectious diseases in the studied group of children (*P* < 0.01), the authors emphasized the importance of including measurements of IgG subclasses in the diagnostic panel [26]. The high incidence of immunoglobulin deficiency compared to healthy individuals was confirmed by recent studies by Diller GP *et al.* (2023) [37]. In a later conventional immunological study (Radford D. *et al.* (1990)) of 66 children with CHDs, 32% of children were diagnosed with IDDs based on all generally accepted criteria for assessing immunity parameters (IgG, IgA, IgM, T-cell immunity): in the majority of patients (53% of 66 children) IDDs concerned changes in IgG type, and in 26 patients (39% of cases), a deficiency of the IgG4 subclass prevailed. The authors found that children with conotruncal defects (tetralogy of Fallot, double-outlet right ventricle, transposition of the great arteries, and truncus arteriosus) are more predisposed to the development of T-cell dysfunction-related immunodeficiency and disorders associated with the IgG subclasses, mainly IgG4, and emphasized that immune disorders and deficiency of immunoglobulins and T-cells were found even in non-syndromic CHD patients. According to the authors, early recognition of IDDs and especially deficiency of IgG subclasses in CHD children can improve the results of antimicrobial and immune disorder therapy with immunoglobulin products [27].

Our early studies based on first- and second-level immunologic tests (WHO, 1983) confirmed that the degree of secondary IDDs is determined by the severity of the circulation disorder and arterial hypoxemia in cyanotic CHDs and is aggravated after surgery, whereas correction of the initial quantitative or functional deficiency of T-cells, B-cells, and natural killer cells before and after cardiac surgery significantly reduces the risk of postoperative purulent-septic and infectious complications [28]. According to some data, at least 30% of CHDs present in combination with genetic syndromes are accompanied by extracardiac abnormalities, with developmental anomalies of the central nervous system, musculoskeletal system, and immunodeficiency [38]. Approximately 13 syndromes have been reported in medical literature in which immunodeficiency and CHDs may coexist. Some genetic syndromes tend to be especially often combined with CHDs, including Down syndrome, congenital asplenia [27], DiGeorge syndrome, 22q11.2 deletion syndrome, and Kabuki syndrome [39-41].

The exact mechanisms underlying the association between immunodeficiency and CHD are not yet fully understood. However, it has been suggested that thymic dysfunction, abnormal lymphocyte differentiation, and chronic inflammation play a key role in this process [42]. A study by Ahmed *et al.* (2022) found that surgical correction of CHD involving partial or complete thymectomy contributes to the development of congenital and adaptive B-cell immunodeficiencies [43]. Some studies have shown that genetic factors underlie the occurrence of CHD and immunodeficiency as comorbidities since identical genetic abnormalities may be found in both conditions. Further research is warranted to better understand the mechanisms and establish the genetic factors underlying and contributing to this association [36, 41, 42]. Thus, IDDs are a relevant problem in children with CHDs: they worsen surgical outcomes and increase the risk of mortality, so early identification and treatment of immunodeficiency in patients with CHDs is vital to improve long-term outcomes. The role of hypoxemia in immune disorders, regardless of the presence of CHD, has been actively studied in recent years. A study by A. A. Kiani *et al.* (2021) was devoted to the potential consequences of reduced oxygen levels and tissue hypoxia in enhancing the expression of Hypoxia-inducible factor 1-alpha (HIF-1 factor), a dimeric protein complex that performs many important functions in immunity. It turned out that the role of HIF in the immune system is very significant and concerns all defense mechanisms, innate and adaptive immunity. The hypoxic response in cells and tissues is coordinated by a group of hypoxia-induced HIFs transcription factors (HIF-1, HIF-2 and HIF-3). HIF-1 is expressed and found in various populations of immune cells, including macrophages, neutrophils, and dendritic cells, as well as in T and B lymphocytes and immune lymphoid cells. HIF-2 expression is also found in various cell types, including endothelial cells and some immune cells, including myeloid and lymphoid cells, which are induced by cytokines in response to hypoxia. This data, in our opinion, largely explains the decrease in various immune defense mechanisms, expressed to a greater extent in cyanotic CHD, discovered in various years by various researchers [44]. Oxygen concentration is closely related to cellular proliferation, division and survival of cellular structures and organelles and is usually supported by homeostatic mechanisms acting at the cellular and systemic levels of organs (tissues) [1-6]. HIF expression can be caused by hypoxia itself, as well as other factors associated with pathological stress, including cancer, inflammation, and bacterial infection.

Adaptation to hypoxia, as a universal mechanism of tissue damage and inflammation, is regulated by hypoxia-induced factors (HIFS). In addition, HIF are central regulators of many innate and adaptive immunological functions, including cell migration, antigen presentation, cytokine and antimicrobial peptide production, phagocytosis, and cellular metabolic reprogramming. A characteristic feature of immune cells is their ability to penetrate and function in tissues with low levels of nutrients and oxygen. In a literature review by Y. Chen and T. Gaber (2021), it was concluded that hypoxia and HIF response have different effects on immune activity in normal and pathological conditions, affecting initiation and cooperation not only in the immune response, but also contributing to the development of immune dysfunction in autoimmune processes [45].

In our opinion, understanding the molecular mechanisms of HIF expression in response to hypoxia explains many immune disorders in CHD and especially in cyanotic CHD with systemic hypoxemia.

In clinical practice, the study of immunity is crucial for the diagnosis and treatment of various diseases. There are many methods available for quantitative and functional assessment of the immune system, including cellular and humoral immunity tests, identification and quantification of immune cell populations and subpopulations, immunoglobulin (Ig) levels, complement levels, and cytokine profiles [30, 33, 46, 47], as well as complex immunohistochemical studies. New, highly effective techniques are used to identify rare genetic mutations that cause immunodeficiency, including genetic technologies for whole exome sequencing and next-generation sequencing [36, 48, 49]. Of the 2,392 infants tested using next-generation sequencing (NGS), 51 infants were diagnosed with seven different types of PIDs, and combined immunodeficiency with associated or syndromic features turned out to be the most common, accounting for 25 of the 51 cases (49%). Fortunately, 35 patients (68.6%) have either been cured or have improved since being diagnosed with PID [48]. In recent years, modern imaging techniques, such as computed tomography (CT) and magnetic resonance imaging (MRI) have been used to identify structural abnormalities in the immune system. These studies can also be used to monitor disease progression and response to treatment [50].

## 5 STUDY OF IMMUNITY AND METHODS OF EXAMINATION

Given the limited informational value of standard laboratory methods, immunologic tests have proven significant as differential diagnosis criteria for inflammatory and non-inflammatory myocardial lesions when deciding on the need for surgery in patients with CHD cardiomegaly syndrome and severe myocardial dysfunction [28, 51]. The choice of the appropriate study method depends on the clinical context and the suspected underlying immunodeficiency.

Over the last 3 decades, TREC and KREC-DNA markers for the development of T- and B-cells-have shown their informative value in the assessment of the function of cellular immunity. Studies have shown that during the maturation of T- and B-cells in the thymus and bone marrow, the cellular receptors are formed through the recombination of genes in the DNA strand, which leads to the creation of a unique region capable of recognizing external agents. During each recombination, a small fragment of the strand is removed, forming a T-cell receptor excision circle (TREC), and kappa-deleting recombination excision circle (KREC) [52]. The discovery of TREC and KREC has opened new avenues for studying the immune system and has led to a better understanding of how genetic material rearranges to generate a diverse set of T-cell receptors able to recognize and respond to a wide range of pathogens [29, 52] by producing antibodies [53-55]. Since their initial discovery, TREC and KREC have been extensively studied, and their use as markers of naive T- and B- cells has become widespread in immunological studies in healthy individuals of various ages and disorders [56, 57]. TRECs are highly sensitive and specific for the early detection of T-cell immunodeficiencies (DiGeorge syndrome, severe combined immunodeficiency, and Wiskott-Aldrich syndrome) [29, 58] and HIV infection, where depletion of naive T-cells, as measured by TREC levels, is associated with disease progression and poor prognosis [54, 55, 59]. In diseases with B-cell immunodeficiency (X-linked agammaglobulinemia and common variable immunodeficiency), KREC values are highly sensitive and specific, and in autoimmune diseases, KREC values correlate with the activity of the process and response to therapy [60, 61]. Research has shown that TREC and KREC quantification is a fast, simple, non-invasive, and inexpensive method that allows the screening of large numbers of newborns in a short period of time [62-67]. These markers have become crucial, up-to-date, highly sensitive, and specific components of screening for primary and secondary immunodeficiencies in newborns [31, 55] as part of other tests and markers of comprehensive neonatal screening to identify a wide range of genetic and metabolic disorders [55, 61-63, 65, 66, 68, 69].

Data on the possibility of TREC and KREC quantification as markers of immunodeficiency in CHDs are limited. Significantly lower TREC and KREC levels in CHD patients compared to healthy people were established, as well as in critical CHDs compared to non-critical ones [57]. TREC and KREC assays have emerged as promising predictors in patients with CHDs. A study conducted by Van Zelm MC *et al.* (2011) confirmed that low TREC and KREC levels correlate with increased mortality in adult patients with CHDs [60]. Similarly, a study by Jiang *et al.* in 2016 showed that low levels of TREC and KREC correlate with an increased risk of infections in children with CHDs [68].

Katie Kennedy *et al.* (2020) conducted a retrospective analysis of TREC levels in children with CHDs aged under 12 months in a Pennsylvania clinic based on medical records of 441 newborns with CHDs. The data were compared with neonatal screening results, types of heart defects, genetic test results, and types of cardiac surgery. In contrast to several studies that indicated false-positive results with neonatal screening, high reliability of TREC results was found (< 1%) for screening for primary severe combined immunodeficiency, which led to the recommendation of further study of the relationship between CHDs and TREC screening in newborns [70].

In a study by D.A. Cheremokhin in 2022, a fairly high prevalence of chromosome 22q11.2 deletion syndrome, also known as DiGeorge syndrome, velocardiofacial syndrome or TBX1 deficiency syndrome, was discovered in the Sverdlovsk Region, up to 1 in 9,895 children. With this syndrome, CHDs were observed in 72% of cases, thymic hypoplasia in 58%, and decreased TREC levels in 49% of cases. Based on post-mortem screening of TREC/KREC levels in 271 children, the authors diagnosed 135 cases of primary immunodeficiencies. Of these, CHDs were observed in 50 cases, *i.e.* in 37% of cases. Studying the children who died of CHD and whose immune-dependent pathology debuted in the first year of life, the author states the importance of studying the molecular genetic aspects of primary immunodeficiencies during preoperative diagnosis from the moment of birth to reduce mortality [36].

Our studies conducted in 200 infants with CHDs in 2020-2022, before the wide use of TREC and KREC screening in Russia, showed a high incidence of decreased TREC levels in children with CHDs (n = 47; 23%), mainly in critical cyanotic CHDs and conotruncal defects. After cardiac surgery, there was a significant increase in the frequency of infectious complications in children with lowered TREC levels compared to children with CHDs and normal TREC values (*P* = .0000, in 36% and 3.6% of observations, respectively) [71].

In contrast to the data on TREC levels, data on the use of KREC levels as a biomarker of IDD in CHD are presented in individual studies, where a significant decrease in KREC levels was found in children with CHDs compared to healthy controls [72, 73]. In addition, KREC levels were positively correlated with thymic size, suggesting thymic dysfunction in patients with CHDs, and impaired B-cell production [72, 74].

## CONCLUSION

Analysis of publications on the high risk of infectious complications of cardiac surgery in children with congenital heart defects and the frequency of immunodeficiency in this category of patients confirms the diagnostic value of TREC and KREC levels. The inclusion of the method in therapeutic strategies or intervention protocols as a preoperative screening in children with congenital heart defects could contribute to improving the results of surgical treatment and reducing mortality.

## AUTHORS’ CONTRIBUTIONS

The authors confirm their contribution to the paper as follows: study conception and design: DE, KA, NI, RT, TM and KI; writing the paper: MB and KD; data collection: AO and data analysis or interpretation: PA and KM. All authors reviewed the results and approved the final version of the manuscript.

## LIST OF ABBREVIATIONS

CHD: Congenital Heart Defect
HIF: Hypoxia-Inducible Factor
HIV: Human Immunodeficiency Virus
IDD: Immunodeficiency Disorder
KREC: K-deleting Recombination Excision Circle
NGS: Next-Generation Sequencing
PID: Primary Immunodeficiency
TREC: T Cell Receptor Excision Circle
